# The Quest for the Holy Grail Of 3D Printing: A Critical Review of Recycling in Polymer Powder Bed Fusion Additive Manufacturing

**DOI:** 10.3390/polym16162306

**Published:** 2024-08-15

**Authors:** Bruno Alexandre de Sousa Alves, Dimitrios Kontziampasis, Abdel-Hamid Soliman

**Affiliations:** 1 Department of Engineering, School of Digital, Technology, Innovation & Business, Staffordshire University, College Road, Stoke-on-Trent, Staffordshire ST4 2DE, UK; bruno.alves@research.staffs.ac.uk; 2 Ford-Werke GmbH, Henry-Ford-Straße 1, 50735 Cologne, Germany; 3 School of Science and Engineering, University of Dundee, Dundee DD1 4HN, UK; 4 Dundee International Institute of Central South University, Central South University, Tongzipo Road, Changsha 410013, China; 5 School of Mechanical Engineering, Faculty of Science and Engineering, University of Leeds, Woodhouse Ln, Leeds LS 29JT, UK

**Keywords:** additive manufacturing, circular economy, polymer, sustainability, 3D printing

## Abstract

The benefits of additive manufacturing (AM) are widely recognised, boosting the AM method’s use in industry, while it is predicted AM will dominate the global manufacturing industry. Alas, 3D printing’s growth is hindered by its sustainability. AM methods generate vast amounts of residuals considered as waste, which are disposed of. Additionally, the energy consumed, the materials used, and numerous other factors render AM unsustainable. This paper aims to bring forward all documented solutions in the literature. The spotlight is on potential solutions for the Powder Bed Fusion (PBF) AM, focusing on Selective Laser Sintering (SLS), as these are candidates for mass manufacturing by industry. Solutions are evaluated critically, to identify research gaps regarding the recyclability of residual material. Only then can AM dominate the manufacturing industry, which is extremely important since this is a milestone for our transition into sustainable manufacturing. This transition itself is a complex bottleneck on our quest for becoming a sustainable civilisation. Unlike previous reviews that primarily concentrate on specific AM recycling materials, this paper explores the state of the art in AM recycling processes, incorporating the latest market data and projections. By offering a holistic and forward-looking perspective on the evolution and potential of AM, this review serves as a valuable resource for researchers and industry professionals alike.

## 1. Introduction

Additive Manufacturing (AM) is a group of methods that are also known as 3D printing, which possess the ability to fabricate and manufacture directly in three dimensions. AM is moving forward at a fast pace, especially considering the industry where the market in this field had increased up to 23% by 2021 [1]. The global market for products and services related to 3D printing was valued at approximately USD 20.37 billion in 2023 [2]. Furthermore, sector projections anticipate that by 2030, this market will soar to an estimated USD 87.2 billion [3], demonstrating a clear trajectory that suggests it will surpass all other manufacturing techniques in the coming decades. In 3D printing, objects are fabricated initially starting from a three-dimensional virtual model that is designed using Computer Aided Design (CAD) software. This model is used to subsequently produce the desired object layer by layer with the use of specialised apparatus called 3D printers or AM printers.

The key advantages of 3D printing are its flexibility in designing, its mass customisation, and its ability to manufacture sophisticated structures. However, a considerable amount of waste is produced as a by-product, when 3D printing is employed for polymers, a fact that is hindering its sustainability and growth. One example, if one considers the Powder Bed Fusion (PBF) category, is the unused thermoplastic powder that remains unutilised when using the Selective Laser Sintering (SLS) technique. It is calculated that for the manufacturing of 10 kg of parts, 23 kg of powder remains unused. This powder is then disposed of without being sintered [4]. Undoubtedly, all this waste material is a cause for concern when generating new builds. Evidently, the necessity and urgent need arises to identify and introduce successful recycling processes.

All manufacturing methods can be divided into: (a) Formative Manufacturing (FM), (b) Subtractive Manufacturing (SM), and (c) Additive Manufacturing (AM).

**Figure 1:**
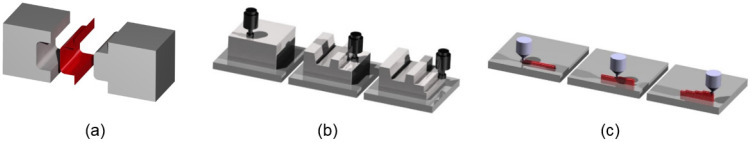
Classification of Manufacturing Techniques. (**a**) Formative Manufacturing (FM), (**b**) Subtractive Manufacturing (SM), and (**c**) Additive Manufacturing (AM).

One can say that formative manufacturing (FM) (Figure 1a) is the process that forms material into a desired shape using a mould (i.e., stamping process [5], high-injection moulding [6], or vacuum forming [7]. This is the favourite and preferred manufacturing method for mass production in industry, mostly due to the production of parts with low cost, speed, and quality consistency of parts with the same characteristic needs.

Contrarily, with subtractive manufacturing (SM), no mould is required (Figure 1b) as in this process the material is removed from a bulk solid, which is typically called block or billet. This technique is used for low-to-mid-amount production, mostly for fabricating uncomplicated geometries, as for example milling of tools or parts.

**Figure 2:**
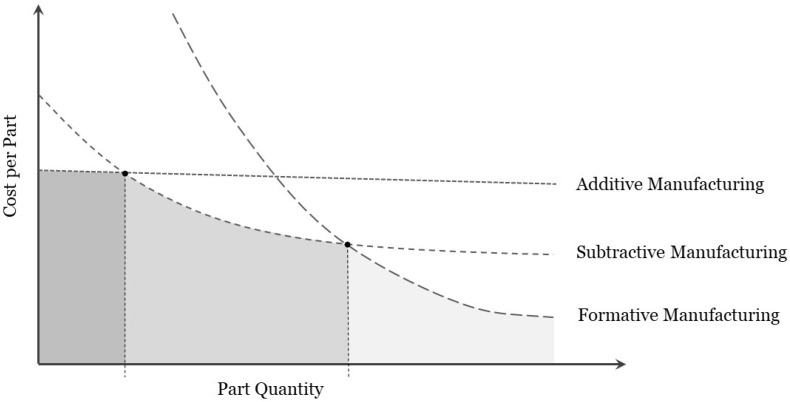
Comparison of production costs per part (standard mass-produced plastic part using the forementioned 3 Manufacturing Techniques).

The newest technique is additive manufacturing (AM) (Figure 1c). This is currently used for low volume production and for the manufacturing of complex geometries, but as mentioned above it is gaining more popularity and is projected to be the dominant manufacturing method in the near future, and is even expected to pave the way for the fourth industrial revolution [8]. Additionally, the cost per part varies significantly depending on the creation process, with additive manufacturing often being more cost-effective for low quantities compared to subtractive manufacturing and formative manufacturing (Figure 2). This economic advantage further underscores the potential of AM to revolutionize manufacturing practices.

**Figure 3:**
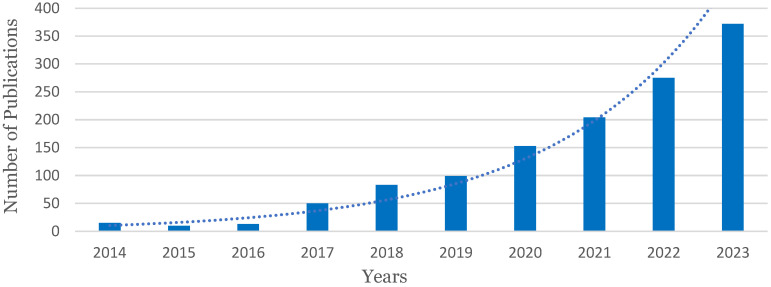
Number of peer-reviewed papers related to recycling materials in AM (Source: Scopus). Papers until end of 2023 (search input: (TITLE-ABS-KEY (recycling) AND TITLE-ABS-KEY (“additive manufacturing”) OR TITLE-ABS-KEY (“3d printing”) OR TITLE-ABS-KEY (“material extrusion”) OR TITLE-ABS-KEY (“material jetting”) OR TITLE-ABS-KEY (“powder bed fusion”) OR TITLE-ABS-KEY (“binder jetting”) OR TITLE-ABS-KEY (“material jetting”)).

Three dimensional printing technology has become more accessible and affordable in recent years, as the starting point to acquire low-cost apparatus has led to an increase in development and innovation in this field, which has subsequently catalysed a dramatic increase in the number of publications in journals about recycling in 3D printing (Figure 3).

### 1.1. Polymers

Polymers are large scale molecules that are derived from the multiplication of monomer units. They are characterised by a significantly higher molecular mass average compared to the monomer itself. Most of the well-known and widely used polymers are constructed via the use of a single monomer. In the case of polyamide-12, which is also known as PA-12, Polylaurolactam, Polylaurinlactam, Poly(12-aminododecanoic acid lactam), or Nylon-12, and is the most widely used polymer in PBF, the consecutive linking of 12-aminododecanoic acids leads to the formation of lengthy chains, which results in the creation of PA-12 [9,10]. When a monomer engages in a chemical reaction with another monomer, it undergoes a transformation into the repeating unit that constitutes the polymer [11]. During the synthesis of PA-12, there is a rearrangement occurring between the carboxylic acid and amine functional groups, which results in the creation of the amide functional group. This newly established amide function serves as the bond that links the two initial molecules. The process repeats itself with multiple iterations during the synthesis, leading to the formation of lengthy chains comprised of repeating units. The culmination of this iterative process yields the macroscopic entity that is known as a polymer [11].

### 1.2. Additive Manufacturing Methods

**Figure 4:**
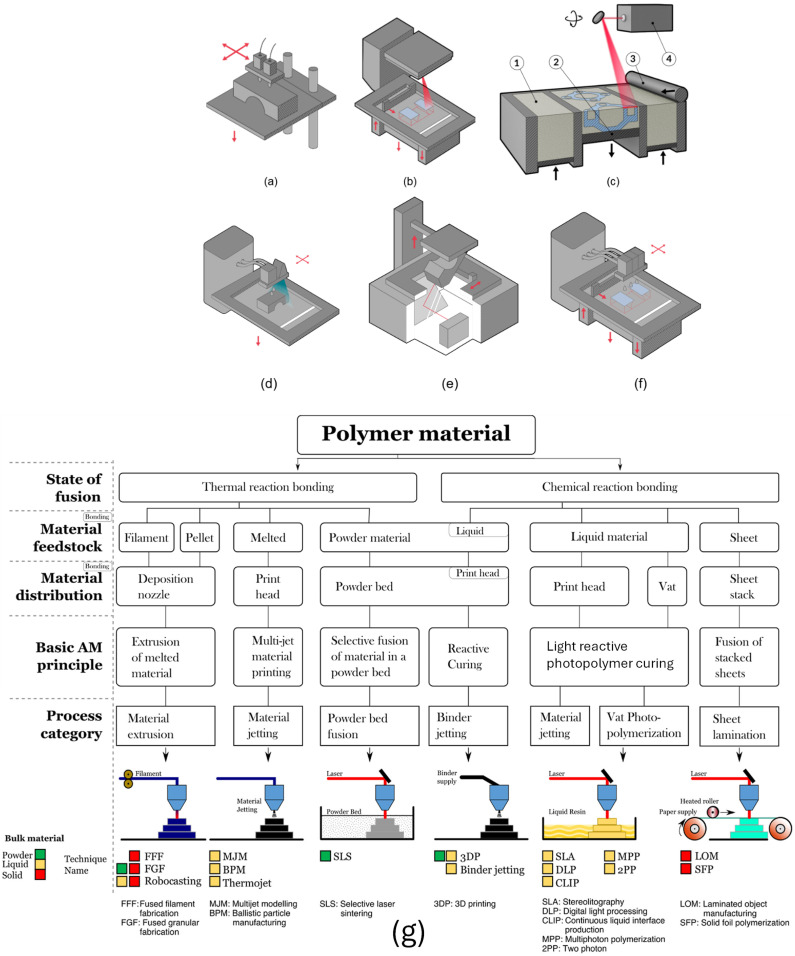
Schematic representation of (**a**) FDM process, (**b**) SLS, (**c**) SLS apparatus components, (**d**) 3DP process, (**e**) stereolithography process, (**f**) binder jetting process, and (**g**) single-operation AM processes that use polymers, modified with permission from [13].

A plethora of AM processes exists, while each year numerous new machines are introduced to the market (Figure 4g), in which based on CAD models, solid and liquid materials can be transformed into three-dimensional objects, i.e., powders (PBF), liquids (stereolithography), or molten solids (Fused Deposition Modelling) normally from filament materials. The above-mentioned processes are also commonly referred to as rapid prototyping/manufacturing/tooling. The initial forming process involves melting down the raw material or starting with a liquid state material, which is subsequently shaped and solidified to create the final object [12].

One very well-known process that falls under this category is Fused Deposition Modelling (FDM) (Figure 4a), where the thermoplastic material is melted and applied in molten form by a heated print head, which gradually manufactures in a layer-by-layer fashion until the final desired object is constructed. The application of each individual layer in a horizontal direction is one of the reasons behind the capability of this method to fabricate complicated morphologies, structures, and sophisticated detailed designs.

There are also processes, such as SLS (Figure 4b,c), where pulverulent material, typically polymeric (Figure 4g) [14], is applied layer by layer using a roller or a blade. Another method is digital light projection (DLP), or 3DP (Figure 4d), which involves selectively curing a liquid polymer material with the use of a locally applied laser or UV light to produce a solid, three-dimensional object [15]. Additionally, stereolithography (SLA) (Figure 4e) uses a laser to cure liquid resin into hardened plastic in a layer-by-layer fashion. In contrast, binder jetting (Figure 4f) involves depositing a liquid binding agent onto a bed of powder material to selectively bind the powder and form solid parts layer by layer.

Figure 4g illustrates the AM processes that use polymers, providing a comprehensive overview of polymer-based AM technologies [13].

It is necessary to mention that PBF refers to a group of AM processes that includes several different techniques, where their common characteristic is the use of a powder bed. One of the most popular under the PBF category is Selective Laser Sintering (SLS). However, it is important to note that not all PBF methods use lasers for sintering. Some other PBF methods use electron beams or other energy sources to fuse powder materials together. Focusing specifically on SLS, the apparatus controls a single or multiple lasers to point at predestined locations in spaces that are precisely defined according to 3D model data; thus, the material is fused together through a high-temperature process to form a solid object. This presents a range of benefits compared to other 3D printing methods, which makes it an attractive option for 3D printing applications in diverse industries. SLS offers the advantage that no support structures are needed, simplifying the printing process and reducing material waste. It is a popular technique in industry due to its versatility, high precision, durability, cost-effectiveness, and scalability.

As illustrated by Figure 4c, the SLS printing process begins when the apparatus spreads out the particulate material (1) (notice arrows that show direction of movement) into an even layer over the building platform (2) using a roller (3). The SLS apparatus chamber is gradually warmed until it nears the material melting temperature, followed by the laser (4) sintering the particles, following the CAD model, then the apparatus building platform (2) is lowered incrementally in accordance with a layer thickness of usually less than 0.12 mm. The procedure is repeated, leading to a layer-by-layer-built process, until the desired complete final object is accomplished. When the fabrication of the solid object finishes and the complete build is cooled to a specific temperature, the platform (2) containing the object is transported to a designated breakout station for further processing. There, the platform is lifted by the operator, and the printed object is removed from the surrounding powder, commonly called “cake”. Most of the powder is manually brushed off during the first stage of the cleaning process. Following the initial cleaning, the object is bead blasted to eliminate any remaining powder [16]. The non-sintered powder is removed after the production process, with the aim of being reused in a successive build.

### 1.3. Challenges and Opportunities for Powder Bed Fusion Sustainability

As discussed previously, PBF, as a group of 3D printing techniques, shows an amazing potential for manufacturing according to the needs and desires of industry. Unfortunately, there are some yet unsurpassed bottlenecks that concern the degradation of the material, and the mechanical, structural stability of the printed object [17]. This significant hurdle in powder bed fusion (PBF), particularly in selective laser sintering (SLS) machines, is the persistence of residual polymeric powder. This challenge arises because typical polymeric materials, once degraded, lose their suitability for reuse in subsequent printer runs [18]. Thermoplastic polymers possess the unique capability to become pliable and mouldable upon heating, subsequently solidifying upon cooling, a property which makes them suitable for use in the AM processes. However, when these polymers undergo degradation, they lose the ability to retain these favourable properties. To ensure an effective mixture for consistent printing results, printer producers typically advise that for each build, the used powder should be combined with a refresh rate of at least 50% new powder [19]. The predominant materials utilized in selective laser sintering (SLS) machines are composites primarily derived from PA-12 and PA-11. Additionally, noteworthy selections for 3D printing include materials such as PA 3200GF [16], that is glass bead-filled PA-12, and DuraForm GF and HST [20], which are a glass-filled and fibre-filled PA-12, respectively [9,10,21].

**Figure 5:**
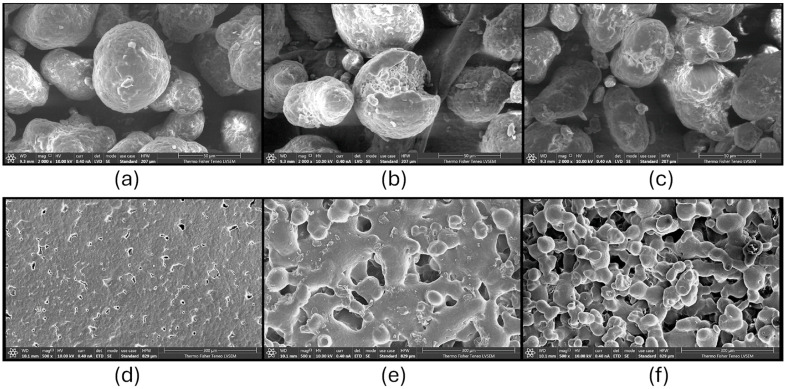

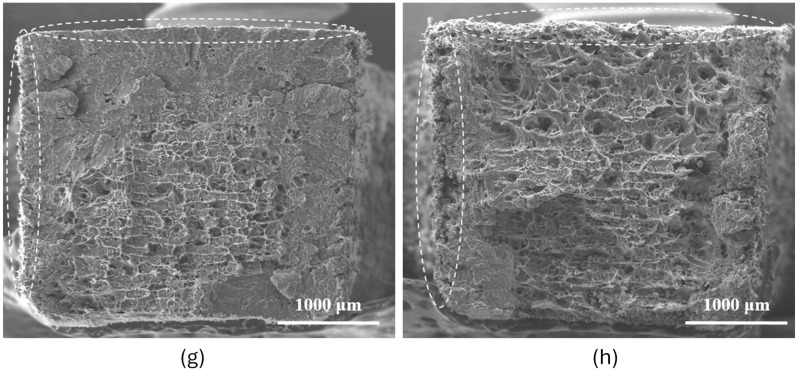

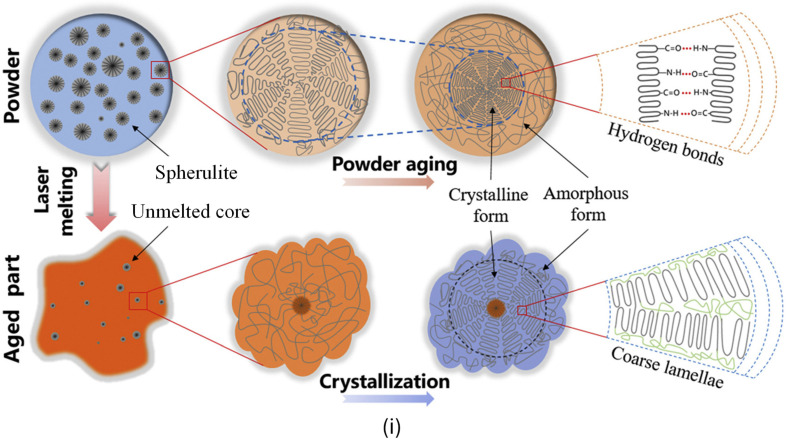
Scanning Electron Microscope (SEM) images for PA-12 powder and part analysis, (**a**) new powder, (**b**) aged powder, (**c**) extremely aged powder, (**d**) components produced with new powder, (**e**) components produced with aged powder, (**f**) components produced with extremely aged powder [19], (**g**) cross-sectional analysis of SLS parts produced from original PA-12, (**h**) cross-sectional analysis of SLS parts produced from 3 times-recycled powder, (**i**) PA-12 powder aging diagram and its SLS process, modified with permission from [21].

Powder degradation poses a significant challenge, as evident from the observed alterations in powder morphology in Scanning Electron Microscope images (Figure 5a–c). Consequently, the suggested practice of employing a 50% ratio (50% new + 50% reused powder) for certain powders has notable technical, economic, and environmental implications for the additive manufacturing (AM) industry [22]. The current average PA-12 powder price is EUR ~100 per kilo [23], which corresponds to a total amount of EUR ~2300 being lost for every 10 kg of printed parts. It is imperative to mention that the increase in the number of times of reusing old powder (refer to Table 1) leads to alterations in the powder’s properties. This, in turn, directly impacts the mechanical properties, such as hardness and impact toughness, of the printed parts [24].

Table 1 showcases that the increase in the number of times of recycling for PA-12 powders corresponds to an increase in the melting point of the recycled material. It can be hypothesised that this increase is due to the reorganisation of the crystalline structure, rather than the degree of crystallinity (Figure 5i). Evidently, the observed decrease in melting enthalpy can lead to an increased secondary sintering of bordering particulates, leading to adverse quality of the surface of printed parts. This phenomenon in numerous cases leads to the formation of different morphologies on the surface of the printed part, as for example the “orange peel” effect [25]. This can be noticed in Figure 5h, where the dimensions and precision of the part that was printed using powder recycled three times is decreased in comparison to the original part that can be seen in Figure 5g. In the case where the recycling exceeds two times of usage, there is an increase in the presence of unmelted powder and the number of pores within the formed samples. This fact leads to a simultaneous reduction in the mechanical properties of the samples, particularly toughness and hardness [26]. When the powder is recycled fewer than two times, there is a minor decrease in impact toughness, as well as an increase in the hardness of the print [26].

**Figure 6:**
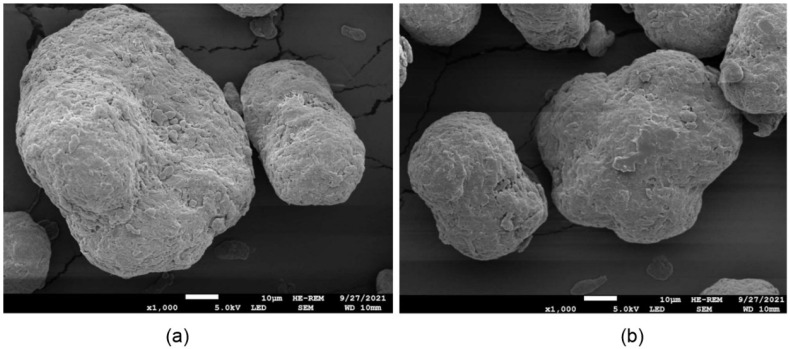
SEM images for (**a**) new powder, and (**b**) aged powder. Notice that there is no clear differentiation between the two at ×1000 magnification [28].

It is essential to dig deeper into the phenomena, and investigate further to decode the mechanisms that are responsible for the mechanical degradation of the printed samples coming from multiply recycled powder. Extensive research on particle size, shape, and distribution has demonstrated that these factors directly influence the mechanical resistance, surface quality, and density of the final product. Additionally, recycled powders may affect spread quality differently compared to virgin powders [27]. P. du Maire et al. identify and document no clear distinction regarding the morphology of the unused powder in direct comparison to the aged one [28]. As can be seen in Figure 6, both new and aged powders exhibit a potato-like particle shape, without significant differences. Further research is therefore necessary, where there will be a use of more samples, in combination with a statistical analysis on the morphological characteristics and the properties of used and recycled 3D printing polymeric powder.

## 2. Sustainability and Circular Economy of Additive Manufacturing

Recently, the need for recycling polymers has been brought into the spotlight of attention, as it directly influences the dramatic increase in plastic pollution, and thus plays a pivotal role in climate change and global warming. It has been showcased that a huge amount of our recyclable material ends up in landfills, dumpsites, and even in our oceans, causing the disequilibrium of ecosystems. New research indicates that less than 10% of the word’s plastic is recycled. Reusing plastic materials does not only reduce human environmental impact but can also decrease the depletion of resources [29].

Similarly, a handful of preliminary studies investigated the recycling of Nylon 12 (PA2200), focusing on Sustainability to Additive Manufacturing (SAM) [11,30,31]. There are some other studies that additionally focused on the circular economy [13]. The key message deriving from these studies is that recycling of 3D printing polymeric powder and cost saving worldwide is essential and necessary from the perspective of sustainability. EOS as one of the world’s leading SLS machine producers, has installed approximately 3000 AM apparatus worldwide. A total of 51 percent are SLS systems [14], and the majority are using Nylon 12, with high apparatus utilisation rates, resulting in a huge “aged Nylon 12” powder excess.

**Figure 7:**
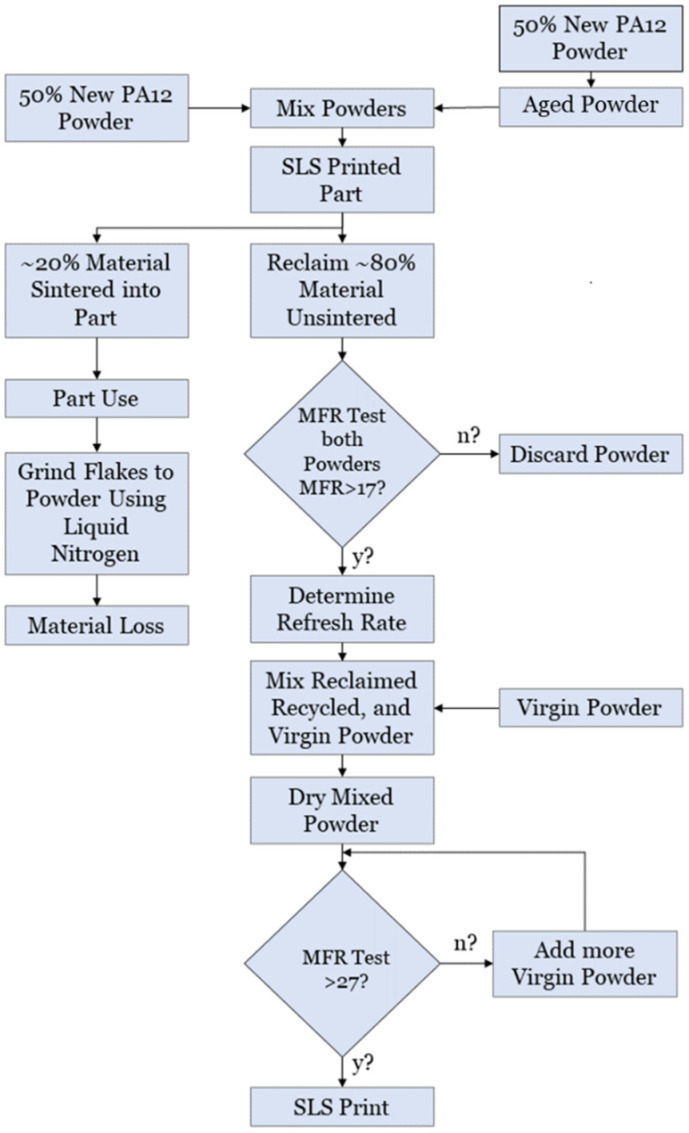
Dotchev and Yusoff’s Powder Recycling Method (PRM). Notice that this process involves recovering both the sintered and the unsintered powder. In the PBF the initial step comprises filling the apparatus with fresh powder, which subsequently undergoes an ageing process, as it is exposed to temperatures in the region of a few tens of °C lower than the standard build temperature, in the areas that the laser is not reaching. When the named particulates are adequately conditioned, they then are mixed with fresh virgin material 1:1, leading the builds from then on to include unsintered powder as well as the material within the sintered part [4].

A common solution that is proposed for this excess powder, which is also referred to as the “aged powder problem”, is to refresh the polymeric powder material with a percentage of new powder to be reused in subsequent prints (Figure 7). This avoids printing issues and the deterioration of the polymer properties in printed parts [32].

**Figure 8:**
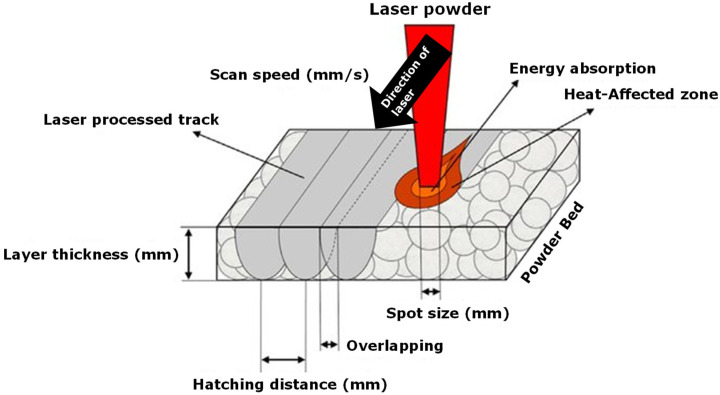
Graphical illustration of the process parameters that are involved during the SLS process and their positioning compared to the powder bed, modified with permission from [33].

In the SLS process, as depicted in Figure 8, the positioning of process parameters relative to the powder bed is crucial. Elevated temperatures and extended exposure times accelerate the degradation of the powder compared to lower temperatures and shorter durations [32].

**Figure 9:**
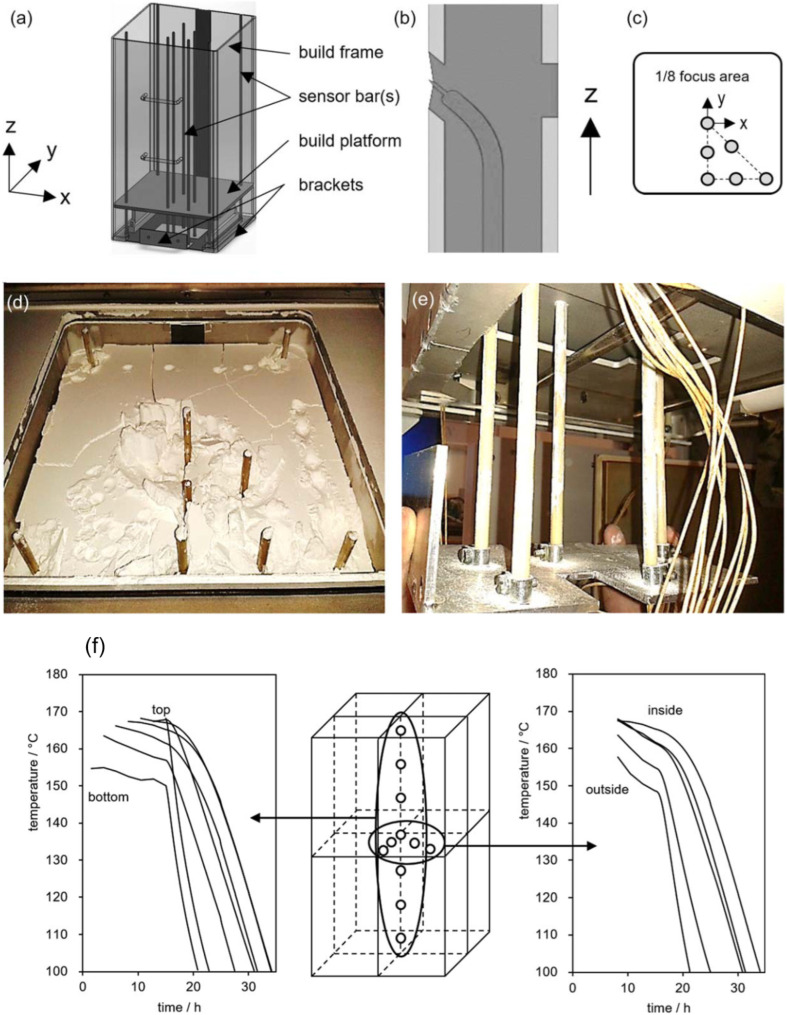
(**a**) Schematic of the apparatus used to measure the temperature in different build chamber positions, (**b**) location of the thermocouples inside the sensor bars, (**c**) location of the sensor bars inside the build chamber, (**d**,**e**) thermocouples and connection assembly inside the SLS chamber, (**f**) results of the thermal mapping during the build (0–15 h) and cooling (>15 h) stages, showing the inside higher temperature and cooling time [35].

Figure 9 shows research where the temperature was measured at various positions within the SLS machine build chamber. By examining the placement of thermocouples within the sensor bars it was observed that areas with higher temperatures experienced increased powder degradation.

Based on this assessment, it can be concluded that the powder ageing process is primarily influenced by the temperature and exposure time parameters [33,34], with the melt flow rate (MFR) index also serving as a highly sensitive indicator of any alterations in the physical properties of the powder. Additionally, MFR provides a relatively quick and cost-effective means of assessing the rate of powder degradation that results from the PBF process, with a particular focus on SLS. Furthermore, it is worth putting special emphasis on the critical role of controlling oxygen concentration within the SLS build chamber. Minimising oxygen levels is imperative, since polyamides, including PA-12, are highly susceptible to oxidation [11].

## 3. Polymeric Material Recycling Methods for 3D Printing

**Figure 10:**
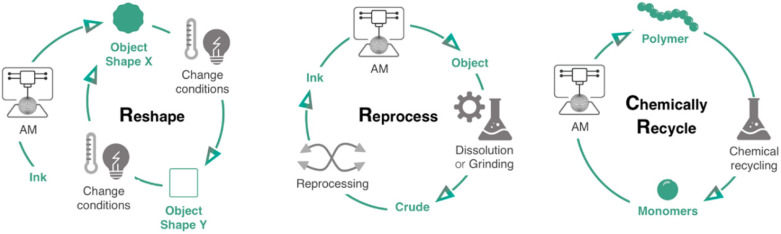
A comprehensive categorisation of approaches for addressing the end of life of 3D printing objects, including the options of reshaping, reprocessing, and chemical recycling [36].

Currently, there are four primary categories of state-of-the-art powder recycling methods for PBF that include heat, chemicals, mechanical pressure, and control of working parameters/powder process. Additionally, printed parts can be recycled by reshaping, reprocessing, or chemical means (Figure 10) [36]. However, the recycling methods are heavily dependent on which AM technique is used. Consecutively, the literature provides numerous different approaches in an effort to recycle material. This review paper aims to provide an insight on the most popular and updated methods for recycling in 3D printing, mostly focusing on methodologies that can provide solutions for PBF AM.

### Use of Filaments

**Figure 11:**
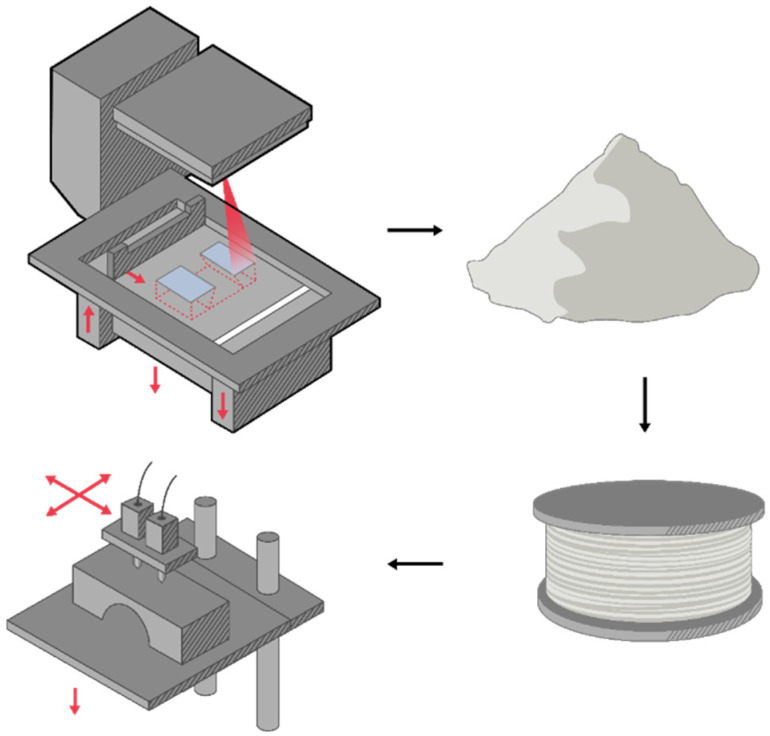
3D graphic representation of recycling SLS aged powder to be used in the FDM process. The aged material is taken from the SLS apparatus and converted into filament, then subsequently it is used to feed the FDM apparatus.

One proposal that poses as a solution for recycling polymeric powder is to use it to produce filaments with an extruder [36]. These filaments can subsequently be used for the FDM process. Figure 11 provides a representation of the recycling process where aged SLS powder is converted into filaments for direct use in FDM printing. To develop these filaments, the used powders are mixed at various percentages with new powder, in an effort to inclusively improve the mechanical characteristic properties that are exhibited by printed parts [36]. A composite approach to recycle polymer powder that is also proposed in numerous papers in the literature involves mixing it with milled carbon fibre (mCF) or other ceramic materials. Using this approach, the findings suggest that the mechanical properties of the printed objects, as for example the tensile modulus, and the impact strength can be enhanced.

As also discussed previously, aged polymeric powder can effectively be recycled for a specific number of cycles. It can subsequently be used in 3D printing, whilst in some cases it can also exhibit improved mechanical properties for a specific number of recycling iterations [37]. However, it has been noted that the effects of recycling the powder to be used in the FDM process results in an increase in porosity, which might not be acceptable for some applications, when considering the quality of the final printed parts. Additionally, it has been noted that these efforts make the total combined process too slow for mass production, turning 3D printing unusable for a plethora of future industrial applications. Evidently, there is a clear need that arises for further study regarding PA-12 and other polymeric particulate materials that are used as powders for AM, and especially for PBF. This is necessary for the quest of the scientific and manufacturing community not only to deepen our understanding of the effect of reusability on the characteristics of polymeric powders, but to understand and decode how this can be materialised both in laboratories as well as in mass manufacturing units.

**Figure 12:**
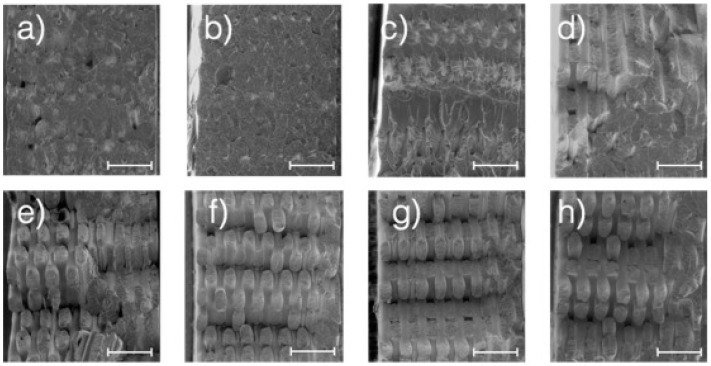
SEM micrographs showcasing the effect of recycling on the mechanical performance of acrylonitrile butadiene styrene (ABS) in FDM. Notice the surfaces with highest fracture stress for (**a**) virgin, (**b**) first round, (**c**) second round, (**d**) third round recycled tensile specimens. See surfaces with the lowest fracture stress for (**e**) virgin, (**f**) first round, (**g**) second round, (**h**) third round recycled tensile specimens. Bars are 1 mm [39].

In contrast to PBF, FDM appears to have more limitations [38]. One very important limitation is the effect of build orientation, which corresponds to the way the part is built and dramatically impacts the build quality. The reason behind this is due to the development of weak connections between layers. Moreover, the slow printing speed, and the risk of build stop for unplanned reasons, may have catastrophic effect on the build and lead to a printing failure. The feasibility of utilising recycled materials for mass production through FDM appears improbable due to the documented effects on mechanical behaviour and structure. As can be seen in Figure 12 for example, one can clearly notice that if the parts are 3D printed with recycled material, a decrease in the mechanical characteristics of the parts is documented and shown in the SEM images in Figure 12a–h, mainly due to dramatic changes in porosity as explained in the literature [39]. This calls for further studies of characterisation, which are deemed necessary to deeply understand the effects of reusability on the characteristics of polymeric materials when used in consecutive FDM printing cycles. Similarly, more studies that clarify the effect of printing cycles are needed for all the materials that are used in SLS, or any other AM method.

## 4. Methods for Recycling to 3D Printing

Most of the literature pertaining to recycling materials to be used in AM revolves around FDM, showcasing significant and beneficial results. In contrast, there exists a discernible gap in comprehensive and diverse research for Powder Bed Fusion (PBF). While notable initiatives have been recorded in suggesting recycling methods to mitigate plastic waste generation (refer to Table 2), there is a recognized necessity for more extensive exploration within the PBF domain. Many of these processes encompass a broad spectrum of steps, with common procedures including material drying, shredding, filament fabrication, and subsequent reuse in 3D printing equipment.

**Figure 13:**
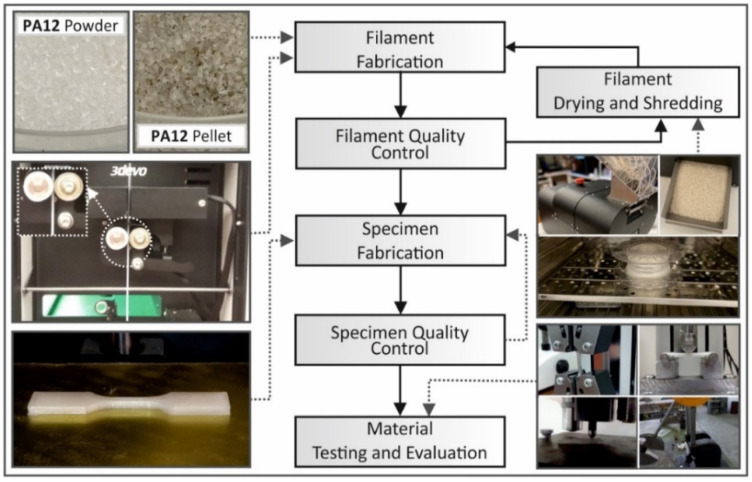
Process diagram of the methodology that is followed for PA-12 in the work from Vidakis et al. (2021), including material drying, shredding, filament fabrication, and reuse of filament in FDM apparatus [37].

Regarding the required steps for recycling, material drying involves removing moisture from the material, which can be achieved through heating or exposure to air. This step is critical as it can aid the prevention of defects in the final build that correspond to the presence of moisture [37]. Subsequently, the shredded material is transformed into filament form (see Figure 13), which involves extruding the material through a small opening in order to create a long, thin strand. The filament fabrication process allows for greater flexibility in the reuse of the material, as it can be suitable for use with a diverse range of 3D printing equipment [120,121]. This filament that has been fabricated is used in a 3D printing apparatus to create new products. The named step allows for a more sustainable use of resources, as the material is reused multiple times. This reduces waste and negative impact on the environment. Overall, employing this type of methodology for recycling 3D printing materials offers a promising solution to the ecological concerns associated with the technology of AM. By implementing the aforementioned methods, one can reduce waste and promote a more sustainable approach to 3D printing.

**Figure 14:**
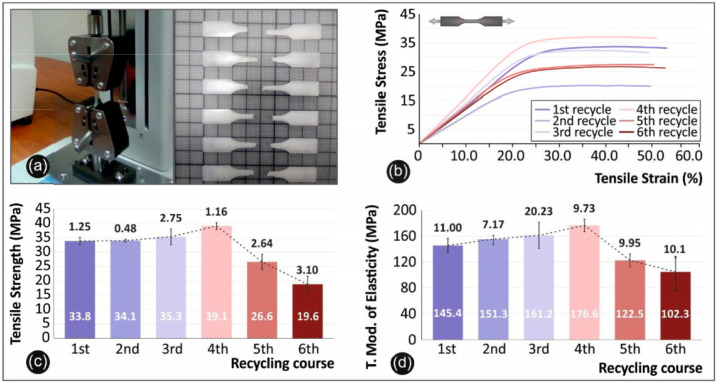

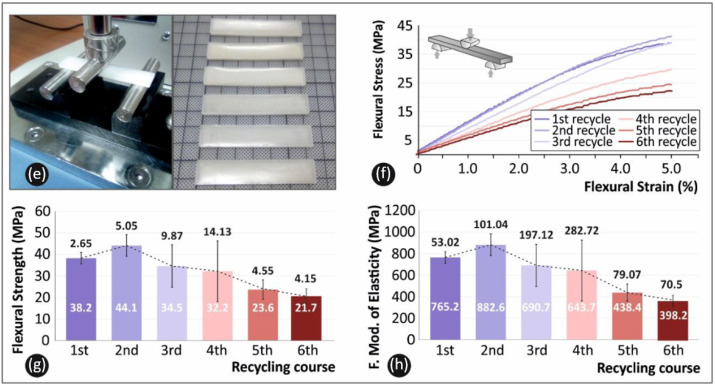

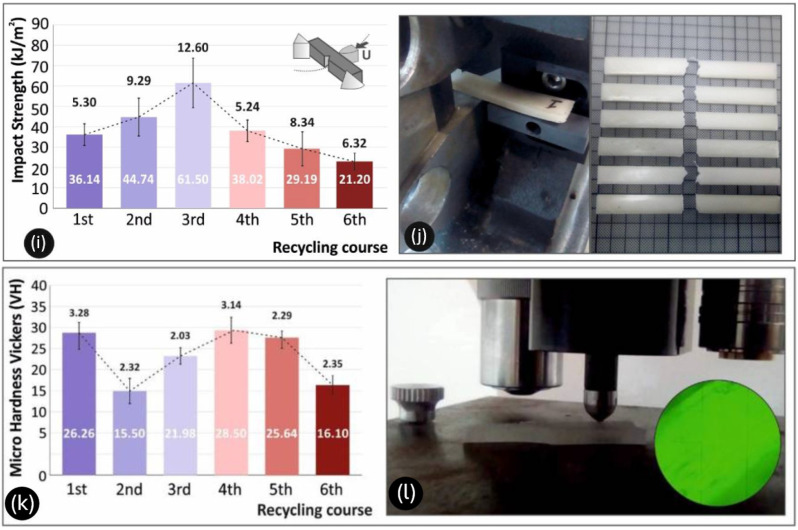
PA-12 recycling experiments: This research involved the recycling of PA-12 material by converting it into PA-12 filament, which was subsequently reused through an FDM apparatus. The study encompassed various tests and analyses, as follows: Tensile Testing. (**a**) Experimental setup of the testing device and post-test specimens. (**b**) Stress–strain curves obtained from the 2nd specimen of each recycling cycle. (**c**) A comparison of average tensile strength values and their respective deviations for each recycling cycle. (**d**) A comparison of average tensile modulus of elasticity values and their associated deviations for each recycling cycle. Flexural Testing: (**e**) Experimental setup of the testing device and post-test specimens. (**f**) Stress–strain curves obtained from the second specimen of each recycling cycle. (**g**) A comparison of average flexural strength values and their respective deviations for each recycling cycle. (**h**) A comparison of average flexural modulus of elasticity values and their associated deviations for each recycling cycle. Impact Testing: (**i**) A comparison of average impact strength values and their associated deviations for each recycling cycle. (**j**) Experimental setup of the testing device and post-test. Micro-Hardness Vickers Testing: (**k**) A comparison of the average Vickers micro-hardness values, including the corresponding calculated deviations, for each recycling cycle. (**l**) Experimental setup of the Micro-Hardness Vickers testing device and post-test specimens [37].

**Figure 15:**
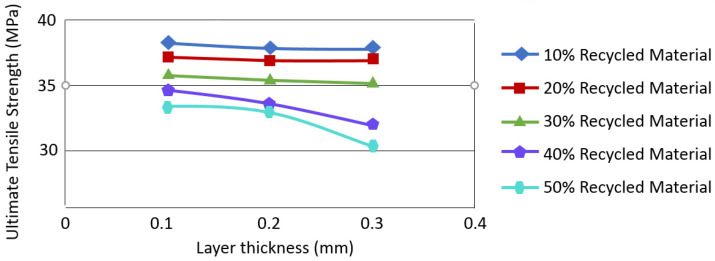
Plot of Polylactic acid (PLA) tensile strength vs. layer thickness, showing the effect of introducing recycled material in AM, modified with permission from [24].

Recycling materials influences the final part’s mechanical behaviour (see Figure 14). In Figure 15, a pattern is depicted illustrating the correlation between the tensile strength and the incremental rise in the proportion of recycled material for polylactic acid (PLA) [24]. However, some polymers are better suited for recycling than PLA. PET (polyethylene terephthalate) has a material recycling rate of 100% comparing to 83% for PLA [44]. There are several research teams that are currently involved in PET and PLA recycling that are identified in the literature (see Table 2).

### Alternative Methods for Recycling

**Figure 16:**
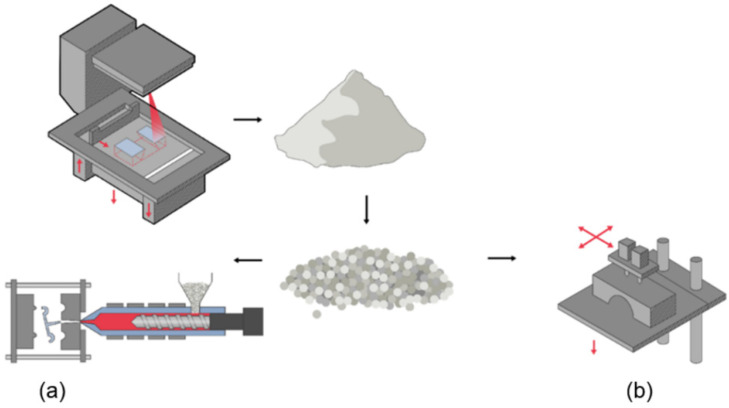
Graphic representation of the steps of a recycling process that uses unfused powder material. The powder is melted and extruded or pressed into small pellets or granules. These granules can then be used as raw material in (**a**) high-injection apparatus or (**b**) through fused granular fabrication to create new products.

Alternative methods of how powders can be recycled and reused are already identified and investigated and can be found in the literature. One example that is commonly met is the use of used PBF powder in the high-injection moulding process (Figure 16a). This recycling process converts the used powder into pellets that are suitable for high-injection moulding mass production facilities [122]. In a recent study, degraded PA-12 powder was utilised from its PBF process having the goal to manufacture commercial vehicle components, including automotive fuel-line clips [123]. There, the researchers recycled material, i.e., 3D printed powders and components, effectively closing the supply chain loop, as they managed to transform them into injection-moulded vehicle parts. This recycling approach can be followed and applied to a plethora of polymeric materials and 3D printing techniques, so for example it can be used for repurposing waste PLA that is generated from FDM [121].

Another method used for recycling is the process that is called Fused Granular Fabrication (FGF) (Figure 16b). FGF is an AM process, in which pellets of polymeric materials are directly used as feedstock, and is considered widely promising by Cruz Sanchez et al. [13], as a method that can enhance the use of recycled polymers in AM. Additionally, another benefit of the proposed method is the widespread availability of low-cost pelletisers that can provide an easy pelletisation apparatus producing pellets of various sizes, so that these could subsequently be fed into an FGF printer [124,125].

**Figure 17:**
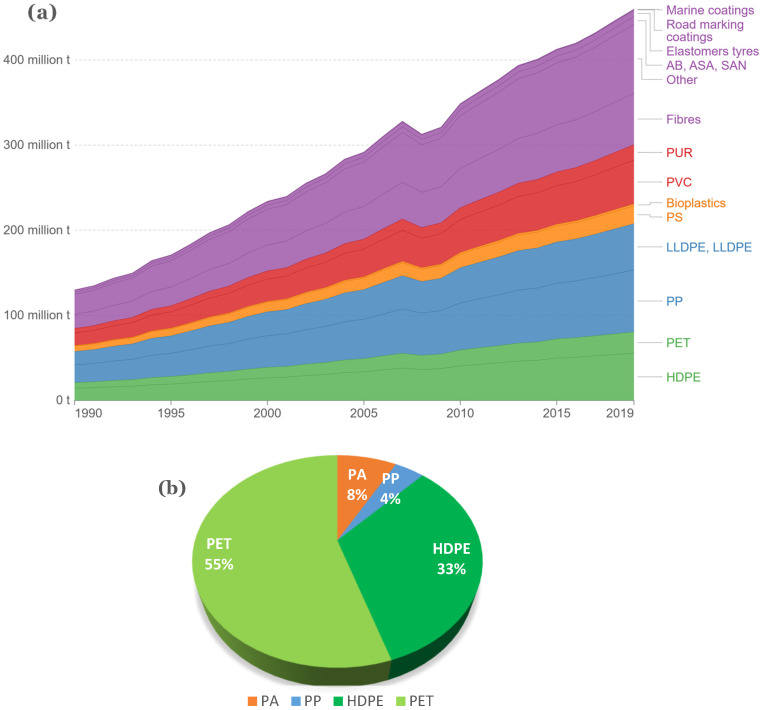
(**a**) Global primary plastic production by polymer, 1990 to 2019, Polymers are colour-coded to indicate recyclability: green for widely recycled, blue for moderately recycled, orange for limited recyclability, red for usually non-recycled, and violet for unknown recyclability [126]; (**b**) the recycled plastics market considering factors impacting the circular economy of plastics for 2020 [37].

The analysis of global plastic usage (see Figure 17a,b) provides an overview of primary plastic production by polymer, categorized based on their suitability for recycling. Polymers are color-coded to reflect their recyclability: green for widely recycled, blue for moderately recycled, orange for limited recyclability, red for typically non-recycled, and violet for unknown recyclability. The polymers identified as the best candidates for recycling include PET (Polyethylene Terephthalate), HDPE (High-Density Polyethylene), LDPE (Low-Density Polyethylene), PP (Polypropylene), PC (Polycarbonate), and ABS (Acrylonitrile Butadiene Styrene) [126]. However, many of the global recycling facilities are confined to PET, PP, HDPE, and LDPE [82]. The polymers used in material extrusion for AM (mostly FDM) are currently limited primarily to ABS, PLA (Polylactic acid), PA (nylon), PC, and PS. Integrating recycling into AM processes can effectively divert these materials from the waste stream, minimise the negative effects on this aspect of AM, and provide a circular economy pathway that is easily applicable [82].

It is important to note and discuss the fact that the recycling of plastics still incurs considerable expense for transportation and collection, as well as the need for a realisation that a substantial amount of energy is necessary for carrying out the named processing of recycling. On top, one needs to consider the costs associated with specialised equipment [82]. Increasing the rate of polymer recycling remains a global challenge that has yet to be adequately addressed and overcome [13,45]. This challenge is further intensified by the industry’s need to manage the “end-of-life” phase for 3D printed objects. The surge in both the adoption of additive manufacturing (AM) and the production of prints over recent years is noteworthy, and as previously highlighted, this upward trajectory is anticipated to escalate at an even higher rate in the near future [36].

In order to save material during the SLS printing process, it is essential to direct attention toward comprehending the dynamics within the build chamber, particularly the behaviour and ageing of the SLS powder. Josypeit and Schmid formulated a model that effectively predicts the ageing pattern within a part cake located in the build chamber. The findings can be used for optimisation of thermal process management, and for innovating more efficient powder-recycling techniques [35]. The polymeric-powder ageing mechanism mainly consists of two conflicting aspects concerning the processability of the polymer itself. Firstly, after three recycling cycles, it was verified that the melting sintering window temperature increases by 1 °C. In parallel, the crystallinity is reduced by ∼6% after three recycling cycles; generally, when crystallinity is reduced, the polymer chains become less organized and more amorphous, reducing tensile strength and stiffness, affecting the mechanical performance of the parts [21].

## 5. Recycling of Materials Used in Additive Manufacturing

**Figure 18:**
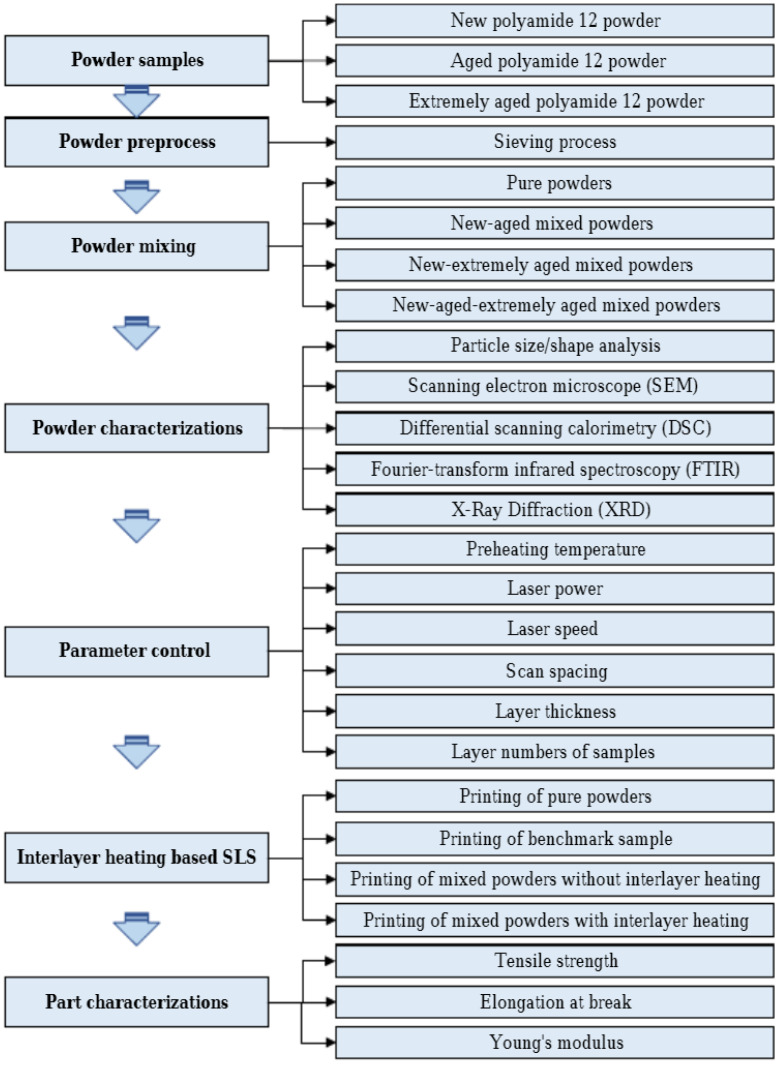
Proposed methodology for maximising reusability of aged PA-12 Powder: This approach encompasses a series of steps, including powder collection, preprocessing, mixing, heating, and thorough characterisation for printed parts reprinted [127].

The recycling of AM materials is an important consideration for achieving sustainability and cost efficiency globally. With this, not only will the companies and 3D printer users avoid contributing to the increase in material being sent to waste, but they can reuse the waste to produce further products, leading to a decrease in manufacturing costs. If companies and users pay special attention to material purity, compatibility, handling, processing techniques, and environmental impact, they will succeed in increasing recycling rates (Figure 18). It is crucial to use high quality materials that are free from impurities and contaminants to avoid affecting the quality and performance of the recycled materials. Without following these methods, in addition to careful handling in order to prevent or to control the degradation that naturally occurs through any type of manufacturing process, the quality and functionality of the prints may be compromised.

A proposed approach that is inexpensive, fast, and simple is to monitor the degradation of the polymeric powder using a dielectric measurement, which is performed by microwave cavity perturbation. This in situ measuring process allows the samples to be extracted from a working SLS apparatus and measured. This is a quality control measurement, which ensures that only degraded material is disposed of, while the other material is fed back into the printer [128].

**Figure 19:**
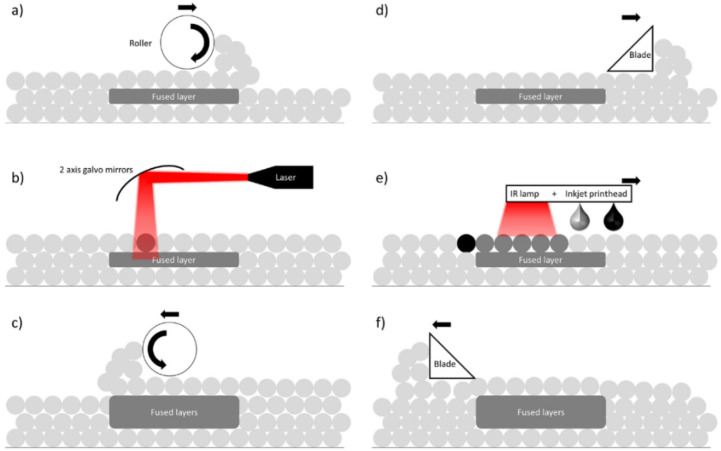
Schematic representation and comparison of SLS (**a**–**c**) versus MJF (**d**–**f**) AM processes [129]. In SLS, (**a**) the roller spreads over the previous layer of the powder, (**b**) the laser selectively heats and fuses the powdered material according to the 3D model, and (**c**) a new layer is added and there is a repeat of the previous steps until the object is completely manufactured. In MJF, (**d**) the blade spreads over the previous layer of the powder, (**e**) the inkjet printing heads deposit a binding agent onto a layer of the powdered material, which the heating element (infra-red lamp) will cause to fuse together in the desired shape, and (**f**) the blade spreads the powder into the next layer, with this step being repeated for each layer of the object until completion of the print.

In recent years, another method within the PBF category has emerged, which is called Multi Jet Fusion (MJF). This technique has led to significant advancements in powder reusability since it allows for parts to be built via layer-by-layer thermal fusion of polymeric particles. Both MJF (see Figure 19d–f) and SLS (see Figure 19a–c) use thermoplastic polymer particulates of ~60–80 μm. The main distinction between MJF and SLS lies in their heat sources. SLS employs a laser to scan and sinter each cross-section. Contrarily, MJF utilises an ink (fusing agent) that is applied to the polymeric powder, which allows for the absorption of infrared light. When in use, an infrared energy source is applied to the build platform, increasing the temperature to the desired window operation, where the fusion of the inked areas takes place. It is evident that MJF can be viewed as an amalgam of the SLS and binder jetting techniques. MJF shares significant similarities with binder jetting, as it utilises a binding agent. However, it is crucial to differentiate and accept MJF as a distinct process that belongs to PBF and clarify that it does not fall under the same category as binder jetting.

In Multi Jet Fusion (MJF), the recovered powder demonstrates an impressive recyclability of 80–85%, surpassing the recycling efficiency of SLS, as indicated by existing literature. As outlined in the white paper titled “Competitive Accelerated Weathering Study Between HP 3D High Reusability PA 12 W and SLS Materials”, the material “3D HR PA 12 W” exhibits greater durability compared to the examined SLS materials. This is evidenced by its ability to maintain stability over an extended period. Specifically, it exhibits colour stability and retains 80–90% of its ductility over time, even under accelerated conditions [130].

Ongoing studies in the literature on restoring the melt rheological properties of the aged polymeric powders, most of which are focused on PA-12, show viable results for the case of acid catalysed hydrolysis [131]. Gallic and phenyl phosphonic acids may facilitate the restoration of the polymer’s rheological properties under low shear stress conditions, which are typical of PBF processing. Phenyl phosphonic acid treatment appears to primarily preserve the polymer’s colour. In both cases, the sintering window of the recycled powder is reduced. In parallel, phenyl phosphonic acid accelerates isothermal crystallisation kinetics, while gallic acid treatment results in a slight reduction in crystallisation time compared to the untreated powder [131]. Another study by Wienmann and Bonten reveals a chemical agent called “chain splitter”, which can be added to the aged powder resulting in the cleaving of the polymer chains at their amide bonds [95]. In this particular study, the cause of the thermal ageing of PA-12 is proposed to be condensation reactions. The use of a chain splitter allows for the molecular effects that stem from post-condensation reactions to be reversed. In principle, this process initiates by breakage of the polymer chains at their amide bonds, which leads to the reduction in the molar mass and molecular weight of the polymer. This adjusts the viscosity of the used powder, in order for it to have comparable values to the ones that typical industrial mixtures have when mixing new and used powder [95]. Depending on the type of chemical agent used as well as its concentration, it is possible to selectively tune the physical chemical properties of the used powder. This ensures the reproducibility and high quality of the named mixtures. During this recycling procedure, used powder attains improved physicochemical properties that allow for tuning of the flow and the sintering process, and furthermore assist in allowing multiple reuses. Consequently, this process enhances the efficiency of the use of material and thus it leads to cost efficiency and cost effectiveness regarding PBF techniques. However, further research is necessary in order to address surface defects observed in complex components treated with certain types of chain splitters, as well as to investigate the reproducibility and the scalability of this method [95].

## 6. Discussion and Conclusions

In this work, considering and reviewing the literature on the recycling of AM, several key areas are thoroughly covered and analysed. On various occasions specific processes are proposed regarding recycling, mainly focusing on small quantities of polymers and unfused polymeric powder/particulate material. One of the most widely used recycling methods proposed in the literature is the use of recycled filaments for FDM. Numerous recent studies have explored the potential for recycling polymeric materials into filament form, but have mainly used ABS, PLA, and PETG. In the literature, the spotlight of attention is shed on the importance of material properties and mostly revolves around purity, in combination with compatibility, handling, and processing techniques, as well as the environmental impact of the recycling of materials for 3D printing.

However, there are still some clearly identifiable knowledge and research gaps that need to be addressed. One of the biggest challenges is the lack of standardisation in the recycling process. This can lead to inconsistent quality and performance issues of recycled materials. Another challenge is the limited availability of recycling facilities and infrastructure, which may impede the broad implementation of recycling in AM. These recycling facilities and infrastructure are a necessity as they are urgently required in order to recycle larger quantities of polymeric materials. This would allow for a “mass recycling” as an answer to mass production and mass generation of waste in industrial processes.

To allow for recycling of AM polymeric materials, there needs to be research that will aid in the development of standardised recycling processes for different types of AM materials. In parallel, it is imperative to improve the availability and accessibility of recycling facilities. The clear need arises for more research on understanding the behaviour of recycled polymeric materials. This includes ageing, the environmental effects on the recycled material during transfer, and during storage the long-term stability of the physical, chemical, physicochemical, and rheological properties of the recycled material, as well as the performance of recycled materials in different applications, to name a few areas.

As the use of SLS technology continues to grow, there is an increasing need for reliable methods of monitoring and understanding the quality of the sintered material. In addition, the effect of the process on the residual material needs to be understood and decoded. Understandably, due to the complex effects and the different scales of phenomena occurring on the surface and in the bulk of the polymeric particulate material during SLS, there are only a few scarce studies investigating the named complex phenomena in the literature. It is necessary to establish a deeper understanding of the effect of the laser on the residual material. However, this can only be realised if one introduces research on particle technology, environmental and polymer chemistry, laser-matter physics and complex phenomena created by lasers on particulate materials (e.g., microplasmas), fluid dynamics, and surface physical chemistry. To avoid this severe complexity, a handful of studies focused solely on the quality of new 3d printed parts, when manufactured with recycled material. However, the quality characterisation of the material in all these studies revolved around the mechanical properties, typically involving the use of a stress–strain apparatus. It is necessary though to validate the quality of the printed parts with the use of many more characterisation methods when using recycled material, as not only the mechanical properties of builds are of interest. It is also imperative that ageing, stability, and durability studies are performed methodically and statistically to deepen our understanding.

Arguably, on a different level, one of the biggest challenges for the recycling in AM is the need for collaboration and cooperation between different stakeholders in the industry, including manufacturers, recyclers, and policymakers. It is important to acknowledge the fact that 3D printing machine manufacturers are also material suppliers for the respective machines, which may create a conflict of interests and limit research on developing machines with lower material consumption rates. More pressure and incentives are needed from organisations and governments to encourage machine manufacturers to understand the importance of achieving higher material-recycling rates. Only through a collective effort can we address the challenges and realise the potential of AM material recycling. This will allow us to transition into sustainable manufacturing and move forward from the traditional subtractive manufacturing processes. If this sustainable manufacturing transition materialises, it will be the biggest step towards moving forward as humanity. This is the biggest and most difficult bottleneck that we face in the current generation. It is evident and clear that we have now started to push towards a total metamorphosis of our current civilisation, as we need it to evolve into a purely sustainable civilisation in order to ensure our future.

## Figures and Tables

**Table 1:** Numerical information regarding the thermal characteristics of PA-12 powders [21].

PA-12 Powder	Peak Melting Point (°C)	Melting Enthalpy (J/g)	Peak Crystallisation Point (°C)	Crystallisation Enthalpy (J/g)	Crystallinity Xc (%)	Sintering Window * (°C)
New	182.37	96.62	141.45	−45.03	46.16%	29.96 (147.15–177.11)
1 time recycled	182.53	95.19	141.94	−44.52	45.48%	29.92 (147.55–177.47)
2 times recycled	182.53	94.02	142.46	−44.71	44.92%	30.31 (147.54–177.85)
3 times recycled	182.87	84.07	142.78	−42.88	40.17%	31.00 (147.33–178.33)
4 times recycled	182.68	80.71	143.24	−42.13	38.56%	30.95 (146.72–177.67)

* The term “sintering window” refers to the temperature range from the start of melting of the material until the onset of crystallisation [21]. Roughly this corresponds to the difference in temperature glass transition temperature (Tg) and crystallization temperature (Tc), measured in degrees Celsius (°C).

**Table 2:** Recycling material in 3D printing as reported in the current literature.

Material Recycled	Method	References
PLA–Poly(lactic acid)	FDM	[24,40,41,42,43,44,45,46,47,48,49,50,51,52,53]
PLA–Poly(lactic acid) PLA–hydroxyapatite (Hap)-chitosan (CS) composite	FDM	[53]
PLLA L900	FDM	[54,55,56]
PLA 2002D	FDM	[57,58]
PLA (ESUN A-1001)	FDM	[59]
PLA/ABS	FDM	[60,61,62]
PLA/ABS/HIPS/PCTPE/Nylon/PC	FDM	[63]
PLA–Poly(lactic acid) + recycled Carbon Fibres (rCFs)	FDM	[64,65,66]
PLA/CLRS-1 c–polylactide/lunar regolith simulant	FDM	[67]
PLA–Poly(lactic acid) and PHB–Polyhydroxybutyrate	FDM	[68]
PLA–Poly(lactic acid) biocomposites filled with CSH–calcined seashell particles	FDM	[69]
ABS–Acrylonitrile–butadienestyrene/with recycled fiber	FDM	[70,71]
PS–polystyrene, ABS–Acrylonitrile–butadienestyrene and PVC polyvinylchloride	FDM	[72]
ABS–Acrylonitrile–butadienestyrene	FDM	[73,74,75,76]
ABS–Graphene composite	FDM	[77]
ABS–Acrylonitrile–butadienestyrene + FA fly ash (waste of coal power plants)	FDM	[78]
HDPE–high-density polyethylene	FDM	[79]
PETG–glycol modified polyethylene terephthalate	FDM	[80]
PETG, PET–Polyethylene Terephthalate	FDM	[81]
PET–Polyethylene Terephthalate, PP–Polypropylene, PS–polystyrene	FDM	[82]
PETG, PET–Polyethylene Terephthalate	FDM	[83]
PETG, PET–Polyethylene Terephthalate, SEBS–styrene ethylene-butadiene-styrene	FDM	[84]
PETG, PET–Polyethylene Terephthalate	FDM	[85,86,87,88,89,90,91,92,93]
PET–Polyethylene Terephthalate + SPF sugar palm fibre	FDM	[94]
PA–Polyamide 12	FDM, SLS	[26,28,29,34,35,37,44,45,48,95,96]
PA–Polyamide composite (Filaments made of carbon fibre with nylon and glass fibre with nylon)	FDM	[97]
PP–Polypropylene	FDM	[83,98,99,100,101]
PP–Polypropylene + glass powder	FDM	[102]
PS–Polystyrene	FDM	[103]
PS–Polystyrene, PVC–Polyvinyl chloride	FDM	[72]
PC–Polycarbonate	FDM	[104,105]
SAG–Styrene-acrylonitrile-glycidyl methacrylate	FDM	[70]
ASA–Acrylonitrile styrene acrylate composites	FDM	[106]
TPU–Thermoplastic polyurethane	FDM	[107]
CAP–Cellulose Acetate Propionate	FDM	[108]
GNP–Graphene nanoparticles and Mn-doped ZnO powders + PVDF–recycled polyvinylidene fluoride pallets	FDM	[109]
Silica + PP/PVA/PLA/PA Polypropylene, polyvinyl alcohol, polylactic acid, and nylon	FDM	[110]
Silica + PLA Poly(lactic acid)	FDM	[111,112]
(PLA composites) Food packages and car dashboards	Composites	[113]
(PLA composites)recycled polylactic acid (PLA) and waste ceramic material from artisanal production	Composites	[114]
(PLA composites) Bio-based composite material consisting of ground mussel shells and alginate	Composites	[115]
Mortar with PET Polyethylene Terephthalate granules	Composites	[116]
Composites–Wind turbine waste	Composites	[117,118]
Mixture comprising 60% granulate tyre waste and 40% of polypropylene (PP)	Composites	[119]

## Data Availability

The authors confirm that there are no original data that are used in this paper, as it is a review paper, and as such there is no Data Availability Statement required.
